# Co-residence with both parents, attitudes toward parents, and psychological difficulties among Chinese secondary school students: indirect associations through social adaptability

**DOI:** 10.3389/fpsyg.2026.1634755

**Published:** 2026-09-30

**Authors:** Zhanghong Li, Jinyin Zhang, Yaning Zhou, Tianye Yang, Zhengzhi Feng

**Affiliations:** 1 Rongchang District Teacher Training School, Chongqing, China; 2 Rongchang Experimental School Affiliated to Southwest University High School, Chongqing, China; 3 Faculty of Psychology, Southwest University, Chongqing, China; 4 Department of Medical Psychology, Army Medical University, Chongqing, China

**Keywords:** adolescents, attitudes toward parents, co-residence, psychological difficulties, social adaptability

## Abstract

**Background:**

This cross-sectional study examined whether co-residence status and students’ attitudes toward parents were associated with psychological difficulties directly and indirectly through social adaptability.

**Method:**

A regional census cluster survey distributed questionnaires to 39,739 students in 20 secondary schools in one district of Chongqing, China. The analytic sample comprised 34,097 valid responses (85.80%). Psychological difficulties were represented by continuous scores from 35 Mental Health Test items covering academic anxiety, self-blame, and impulsivity. Two separate observed-variable path models were estimated with school-level clustering, maximum likelihood estimation, 5,000 cluster-bootstrap draws, and adjustment for sex and grade.

**Results:**

More positive attitudes toward parents were associated with fewer psychological difficulties through social adaptability (standardized indirect association = −0.148, bias-corrected 95% CI [−0.170, −0.129]); the total association was −0.196 [−0.228, −0.162]. Compared with students living with both parents, students living with others showed a small positive indirect association with psychological difficulties (0.034 [0.022, 0.050]) and a small total association (0.049 [0.033, 0.070]).

**Conclusion:**

The findings were compatible with indirect associations through social adaptability, but the cross-sectional design does not establish temporal or causal mediation. Co-residence findings should be interpreted cautiously because the retained dataset did not preserve the original nonparental living-arrangement categories.

## 1 Introduction

Psychological difficulties during adolescence affect learning, relationships, and daily functioning. Population studies in different settings have documented substantial adolescent mental health needs (Wagner et al., 2017), and research using the Mental Health Test (MHT) in Chongqing has identified academic anxiety and self-blame as relevant concerns among school-age students (Ning et al., 2013). These difficulties develop within social contexts. Bronfenbrenner’s bioecological framework places the family in the adolescent’s immediate developmental environment while also recognizing the roles of schools, communities, and broader socioeconomic conditions (Bronfenbrenner, 2005).

Co-residence with parents is one observable feature of the family context, but it is not a complete measure of family structure or functioning. In China, parental migration and grandparent care make the distinction especially important. A systematic review and meta-analysis reported higher average risks of several mental health problems among children left behind by migrant parents, although study quality and effect estimates varied (Fellmeth et al., 2018). Chinese studies have also linked parental migration with psychosocial difficulties and school engagement (Tang et al., 2018; Wen and Lin, 2012). Qualitative evidence further indicates that the meaning of parental absence depends on parent–child separation, caregiver support, poverty, divorce, and the health of grandparent caregivers (Zhao et al., 2018). Therefore, a binary co-residence indicator cannot identify why an arrangement exists or how a student experiences it.

Adolescents’ subjective evaluations of their parents may convey information that household composition does not. Family cohesion and adolescents’ perceptions of family functioning have been associated with depressive symptoms (Cumsille and Epstein, 1994), and family communication has been linked to adolescent mental health through school-related and behavioral correlates (Deniz et al., 2024). The present survey, however, assessed attitude toward parents with one global item. This item may reflect affection, conflict, current mood, or socially desirable responding. It is consequently described as self-reported attitude toward parents, not as family emotional quality, attachment, cohesion, or communication.

Social adaptability refers here to students’ reported capacity to adjust to changing social and learning situations and to interact effectively in unfamiliar or demanding contexts (Zheng, 1999). Family-related experiences may be associated with opportunities, support, and confidence relevant to adaptation. In turn, greater adaptability may coincide with fewer difficulties related to anxiety, self-blame, and impulsivity. Studies of left-behind children have linked parent–child relationships to mental health through personal psychological resources (Mordeno et al., 2019), and research with South Korean adolescents has reported indirect associations between school or parental factors and suicidality through psychological factors (Park et al., 2025). These findings support testing an indirect-association model, although they do not establish social adaptability as a causal mechanism in the present sample.

Indirect associations estimated from cross-sectional data require particular caution. The product of two regression paths can describe whether observed covariances are compatible with a proposed pathway, but it cannot establish temporal ordering. Cross-sectional estimates can also differ from the longitudinal process they are intended to represent (Maxwell and Cole, 2007). The current analysis therefore uses path analysis as a descriptive model of direct and indirect statistical associations.

This study examined two family-related indicators separately in a large regional sample of Chinese secondary school students. We expected more positive attitudes toward parents to be associated with higher social adaptability and fewer psychological difficulties. We also expected living with people other than both parents to be associated with lower social adaptability and greater psychological difficulties. For each predictor, we tested whether the data were compatible with an indirect association through social adaptability after adjustment for sex and grade. We did not treat the two indicators as validated components of a single structure-emotion construct, and we did not formally compare their indirect associations.

## 2 Method

### 2.1 Participants and sampling design

From September to November 2023, a region-wide census cluster survey was conducted among students in 20 regular secondary schools in one district of Chongqing, China. The participating schools included junior, senior, and combined secondary schools. A total of 39,739 questionnaires were distributed. Questionnaires identified as invalid during data cleaning, including those with excessively short completion times, were excluded. The archived study records available for this analysis did not specify the numerical completion-time threshold or retain a breakdown of the exclusion reasons. The final analytic sample comprised 34,097 students, corresponding to a valid-response rate of 85.80%.

The sample included 16,090 male students (47.19%) and 18,007 female students (52.81%). Students were in Grades 7–12; mean grade level was 9.33 (SD = 1.65). The 20 school clusters had a mean size of 1,704.85 students and ranged from 2 to 5,557 students. Table 1 summarizes the available sample and study-variable characteristics.

**Table 1:** Sample and study variable characteristics.

Characteristic	Category or statistic	*n* or *M*	% or SD
Analytic sample	Valid questionnaires	34,097	85.80% of 39,739
Schools	School clusters	20	*M* size = 1,704.85
Sex	Male	16,090	47.19%
	Female	18,007	52.81%
Grade	Grades 7–12	*M* = 9.33	SD = 1.65
Co-residence	Living with both parents	–	63.31%
	Living with others	–	36.69%
Attitudes toward parents	Range 1–5	*M* = 4.29	SD = 0.88
Social adaptability	Range −40–40	*M* = 1.05	SD = 13.88
Psychological difficulties	Range 0–35	*M* = 16.19	SD = 7.95

Co-residence percentages are based on the retained binary variable (0 = living with both parents; 1 = living with others). Grade was entered as a continuous covariate in the final Mplus analyses.

### 2.2 Procedure and ethical approval

Questionnaires were administered electronically during regular class time under standardized instructions. Trained psychology graduate students or school mental health teachers supervised data collection. Responses were anonymous and were used only for research. The study followed the Declaration of Helsinki and was approved by the Ethics Committee of the Army Medical University (Approval No. IEC-C[B013J-02-J.02]). Written informed consent was obtained from legal guardians, and written assent was obtained from participating students.

## 3 Measures

### 3.1 Psychological difficulties

The MHT was developed for psychological screening among Chinese primary and secondary school students (Song and Chen, 1999; Zhou, 1991). The full instrument contains 100 items and eight symptom-related content domains. Previous research has examined the MHT structure and item revision in secondary school samples (Zheng et al., 2004). The regional survey used 35 MHT items: 15 academic-anxiety items, 10 self-blame items, and 10 impulsivity items. These items were the only MHT domains administered in the original survey; the present analysis does not describe their sum as comprehensive mental health or as a validated short-form MHT.

Items were scored Yes = 1 and No = 0. The 35-item total ranged from 0 to 35, with higher scores indicating greater difficulties related to academic anxiety, self-blame, and impulsivity. Although the MHT manual provides a separate reference score of 8 for each subscale, a validated cutoff for the sum of these three selected subscales has not been established. The total was therefore analyzed only as a continuous score; no overall prevalence estimate or binary outcome analysis was conducted. In this sample, Cronbach’s *α* was 0.907 for the 35-item total, 0.851 for academic anxiety, 0.835 for self-blame, and 0.730 for impulsivity.

### 3.2 Social adaptability

Social adaptability was assessed with the 20-item Social Adaptability Diagnostic Scale described by Zheng (1999). The response options were Yes, Uncertain, and No. For odd-numbered items, responses were scored −2, 0, and 2, respectively; for even-numbered items, responses were scored 2, 0, and −2, respectively. All items were thus oriented so that higher values indicated greater social adaptability. Item scores were summed to produce a continuous total from −40 to 40.

The manual describes interpretive score bands, but their normative applicability to contemporary secondary school students has not been established. Accordingly, the present analyses did not classify students into adaptability categories. The measure has been used in more recent Chinese adolescent samples (Aricha, 2024; Li, 2022), although such use does not substitute for formal validation in the present population. In this sample, Cronbach’s *α* was 0.793. The Kaiser Meyer Olkin measure was 0.890, and Bartlett’s test of sphericity was significant, *χ*^2^(190) = 129,610.197, *p* < 0.001, indicating that the inter-item correlation matrix was factorable.

### 3.3 Family-related indicators

Attitudes toward parents were measured with one item: “Overall, what is your attitude toward your parents?” Responses ranged from 1 (strongly dislike) to 5 (strongly like), with higher scores indicating a more positive self-reported attitude. The item was not interpreted as a measure of parent–child relationship quality, family emotional climate, attachment, or cohesion.

Co-residence was originally assessed with four response options: living with both parents, paternal grandparents, maternal grandparents, or others. The retained deidentified analytic file preserved only a derived binary variable coded 0 for living with both parents and 1 for living with others. The individual-level four-category responses could not be recovered for the present revision. The analysis therefore contrasts co-residence with both parents against a heterogeneous combined group and cannot estimate associations for specific nonparental arrangements.

### 3.4 Covariates

Sex was coded 0 for male and 1 for female. Grade ranged from 7 to 12 and was entered as a continuous covariate. No other demographic, socioeconomic, or family-process covariates were available in the retained dataset.

## 4 Data analysis

Descriptive statistics, scale reliability, and bivariate correlations were obtained with IBM SPSS Statistics Version 26.0. School-level intraclass correlation coefficients (ICCs) for psychological difficulties and social adaptability were estimated from unconditional two-level models. Because class identifiers were not retained, school was the finest available clustering variable.

Two separate observed-variable path models were estimated in Mplus Version 8.3 (Muthén and Muthén, 1998–2017). In the first model, attitudes toward parents were the predictor; in the second, binary co-residence status was the predictor. Social adaptability was the intervening variable and the continuous 35-item psychological-difficulty score was the outcome in both models. Sex and grade predicted both social adaptability and psychological difficulties. The equations for each model were:


$$M_{i}=\alpha_{0}+aX_{i}+\gamma ₁\mathrm{Se}x_{i}+\gamma ₂\text{Grad}e_{i}+\varepsilon {₁}_{i}$$

$$Y_{i}=\beta_{0}+c^{\prime }X_{i}+bM_{i}+\delta ₁\mathrm{Se}x_{i}+\delta ₂\text{Grad}e_{i}+\varepsilon {₂}_{i}$$

Here, X denotes the family-related predictor, M denotes social adaptability, and Y denotes psychological difficulties. The indirect association was calculated as ab, the direct association as c′, and the total association as c′ + ab (Wen and Ye, 2014).

Both models used TYPE = COMPLEX with school as the cluster variable, maximum likelihood estimation, and 5,000 cluster-level bootstrap draws. All requested bootstrap draws were completed. We report unstandardized coefficients, bootstrap standard errors, standardized coefficients (STDYX), and bias-corrected 95% bootstrap confidence intervals. All model variables had complete data (covariance coverage = 1.000), so no missing-data procedure was required for these analyses. Each recursive model was just identified; global fit statistics such as *χ*^2^, CFI, TLI, and RMSEA were therefore unavailable or uninformative and were not used to evaluate model fit.

## 5 Results

### 5.1 Descriptive statistics and school-level clustering

The mean psychological-difficulty score was 16.19 (SD = 7.95; observed range = 0–35), and the mean social-adaptability score was 1.05 (SD = 13.88; observed range = −40 to 40). The mean attitude-toward-parents score was 4.29 (SD = 0.88). Overall, 63.31% of students lived with both parents and 36.69% lived with others. Descriptive statistics and correlations for the two analytical models are shown in Table 2.

**Table 2:** Descriptive statistics and bivariate correlations for the two analytical models.

Variable	*M*	SD	1	2	3	4	5
Panel A Attitudes toward parents model							
1. Psychological difficulties	16.188	7.952	–				
2. Social adaptability	1.051	13.878	−0.512	–			
3. Attitudes toward parents	4.286	0.881	−0.206	0.310	–		
4. Sex	0.528	0.499	0.178	−0.178	−0.061	–	
5. Grade	9.331	1.648	−0.056	−0.097	0.000	0.057	–
Panel B Co-residence model							
1. Psychological difficulties	16.188	7.952	–				
2. Social adaptability	1.051	13.878	−0.512	–			
3. Co-residence status	0.367	0.482	0.048	−0.063	–		
4. Sex	0.528	0.499	0.178	−0.178	−0.015	–	
5. Grade	9.331	1.648	−0.056	−0.097	−0.032	0.057	–

*N* = 34,097. Sex was coded 0 = male and 1 = female. Co-residence status was coded 0 = living with both parents and 1 = living with others. Correlations are reproduced from the final Mplus sample statistics; inferential emphasis is placed on effect magnitude and confidence intervals rather than p values.

The school-level ICC was 0.020 for psychological difficulties and 0.018 for social adaptability. Although these values were small, the large and uneven school clusters could still affect uncertainty estimates; all path analyses therefore used school-level cluster resampling. Psychological difficulties correlated −0.512 with social adaptability, −0.206 with attitudes toward parents, and 0.048 with the binary co-residence indicator. These bivariate associations differed substantially in magnitude, illustrating why statistical significance alone is insufficient in this large sample.

### 5.2 Attitudes toward parents model

More positive attitudes toward parents were associated with higher social adaptability, *B* = 4.741, bootstrap SE = 0.246, bias-corrected 95% CI [4.280, 5.240], *β* = 0.301. Higher social adaptability was associated with fewer psychological difficulties after adjustment for attitudes, sex, and grade, *B* = −0.281, bootstrap SE = 0.006, 95% CI [−0.292, −0.270], *β* = −0.491. The direct association between attitudes toward parents and psychological difficulties was also negative, *B* = −0.431, bootstrap SE = 0.066, 95% CI [−0.545, −0.288], *β* = −0.048.

The indirect association through social adaptability was −1.334, bootstrap SE = 0.086, 95% CI [−1.517, −1.180], with a standardized estimate of −0.148 [−0.170, −0.129]. The total association was −1.765, bootstrap SE = 0.139, 95% CI [−2.034, −1.491], *β* = −0.196 [−0.228, −0.162]. The model explained 12.9% of the variance in social adaptability and 28.4% of the variance in psychological difficulties. Table 3; Figure 1 present the full effect estimates.

**Table 3:** Direct indirect and total associations from the cluster-bootstrap path models.

Path or association	*B*	Boot SE	95% BC CI	*β*	95% BC CI	*p*
Attitudes toward parents model						
a: Attitude → adaptability	4.741	0.246	[4.280, 5.240]	0.301	[0.275, 0.328]	<0.001
b: Adaptability → difficulties	−0.281	0.006	[−0.292, −0.270]	−0.491	[−0.522, −0.459]	<0.001
c′: Direct	−0.431	0.066	[−0.545, −0.288]	−0.048	[−0.061, −0.031]	<0.001
ab: Indirect	−1.334	0.086	[−1.517, −1.180]	−0.148	[−0.170, −0.129]	<0.001
c: Total	−1.765	0.139	[−2.034, −1.491]	−0.196	[−0.228, −0.162]	<0.001
Co-residence model						
a: Status → adaptability	−1.959	0.389	[−2.818, −1.298]	−0.068	[−0.096, −0.047]	<0.001
b: Adaptability → difficulties	−0.289	0.006	[−0.301, −0.278]	−0.505	[−0.539, −0.469]	<0.001
c′: Direct	0.238	0.081	[0.075, 0.385]	0.014	[0.005, 0.023]	0.003
ab: Indirect	0.567	0.115	[0.373, 0.827]	0.034	[0.022, 0.050]	<0.001
c: Total	0.805	0.157	[0.545, 1.149]	0.049	[0.033, 0.070]	<0.001

*N* = 34,097 students nested in 20 schools. Models used maximum likelihood estimation, TYPE = COMPLEX, and 5,000 cluster-bootstrap draws. BC CI, bias-corrected bootstrap confidence interval; Boot SE, bootstrap standard error; *β*, STDYX standardized coefficient. Both models adjusted the mediator and outcome for sex and continuous grade. Co-residence status was coded 0 = living with both parents and 1 = living with others. For the attitude model, *R*^2^ = 0.129 for social adaptability and 0.284 for psychological difficulties. For the co-residence model, *R*^2^ = 0.044 and 0.282, respectively.

**Figure 1:**
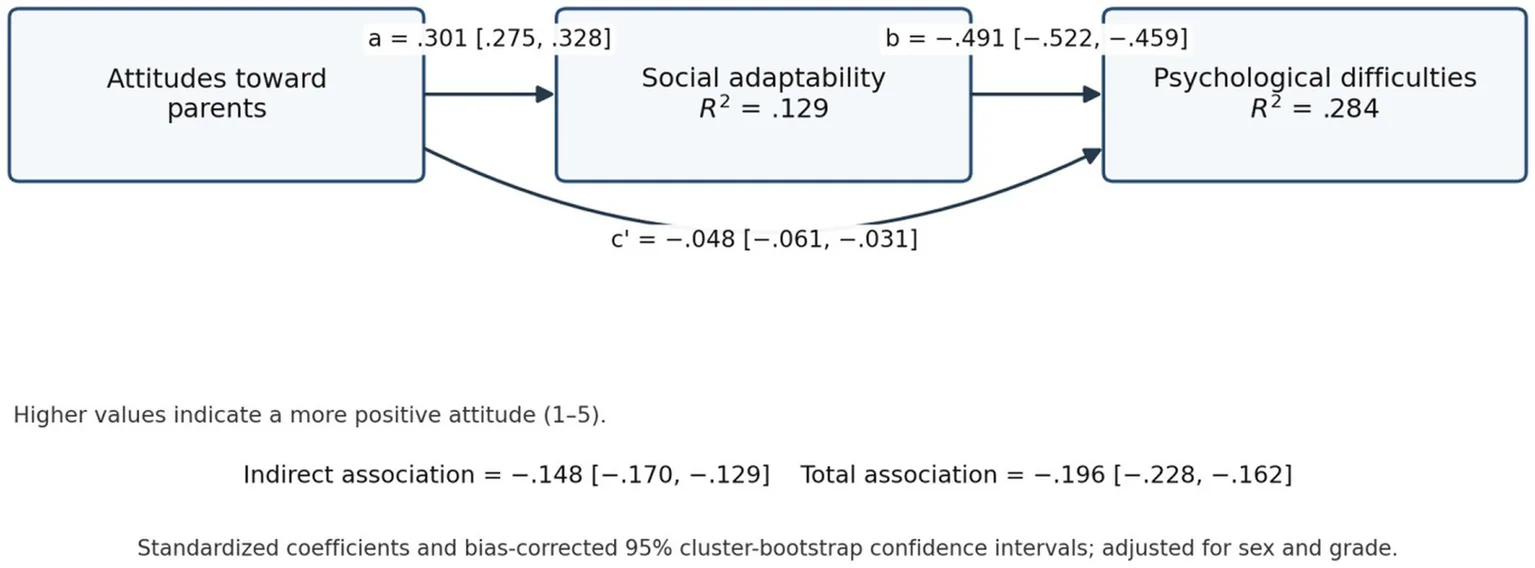
Indirect-association model for attitudes toward parents. All variables were observed. Coefficients are standardized (STDYX); values in brackets are bias-corrected 95% cluster-bootstrap confidence intervals. The model adjusted both endogenous variables for sex and grade.

### 5.3 Co-residence model

With co-residence coded 0 for living with both parents and 1 for living with others, living with others was associated with lower social adaptability, *B* = −1.959, bootstrap SE = 0.389, bias-corrected 95% CI [−2.818, −1.298], *β* = −0.068. Higher social adaptability was associated with fewer psychological difficulties, *B* = −0.289, bootstrap SE = 0.006, 95% CI [−0.301, −0.278], *β* = −0.505. The adjusted direct association between living with others and psychological difficulties was positive but very small, *B* = 0.238, bootstrap SE = 0.081, 95% CI [0.075, 0.385], *β* = 0.014, *p* = 0.003.

The indirect association through social adaptability was 0.567, bootstrap SE = 0.115, 95% CI [0.373, 0.827], with a standardized estimate of 0.034 [0.022, 0.050]. The total association was 0.805, bootstrap SE = 0.157, 95% CI [0.545, 1.149], *β* = 0.049 [0.033, 0.070]. The model explained 4.4% of the variance in social adaptability and 28.2% of the variance in psychological difficulties (Table 3; Figure 2). The standardized total, direct, and indirect associations were small.

**Figure 2:**
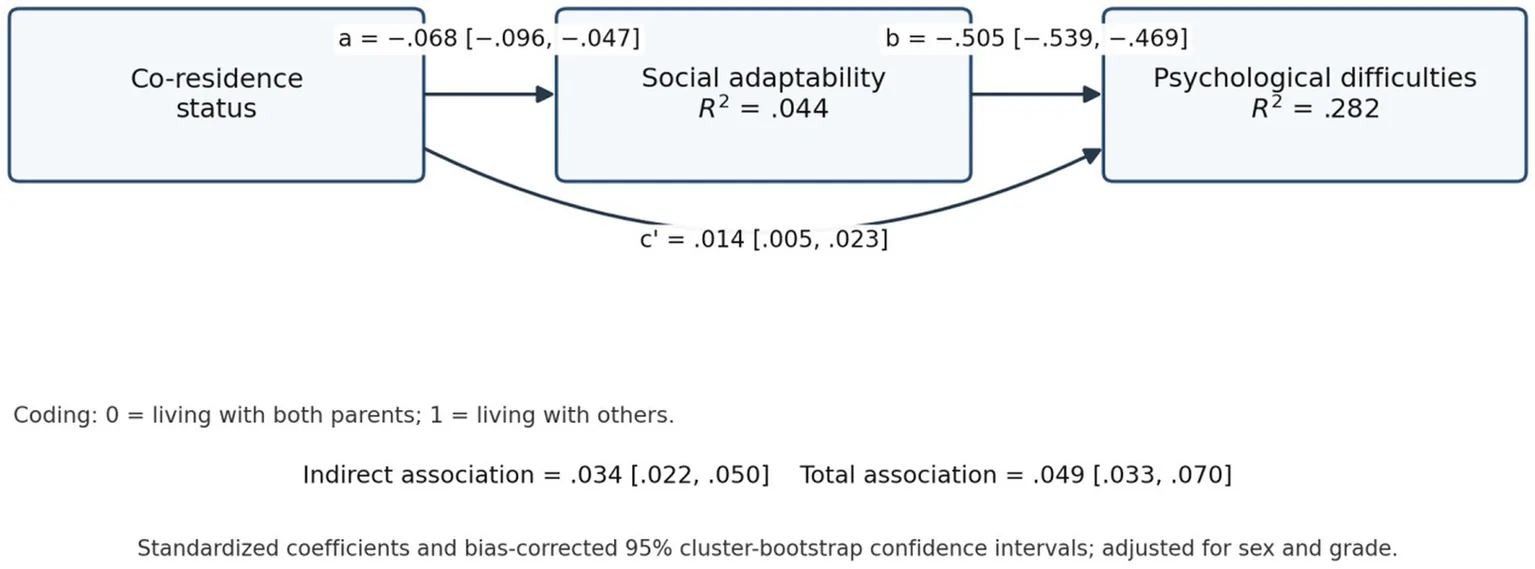
Indirect-association model for co-residence status. All variables were observed. Coefficients are standardized (STDYX); values in brackets are bias-corrected 95% cluster-bootstrap confidence intervals. Co-residence was coded 0 = living with both parents and 1 = living with others. The model adjusted both endogenous variables for sex and grade.

No mediation proportions were calculated because such ratios can be unstable when the total association is small. The two indirect associations were estimated in separate models and were not formally compared; their numerical values should not be interpreted as evidence that one pathway was statistically stronger than the other.

## 6 Discussion

### 6.1 Summary of findings

This study examined two narrowly defined family-related indicators in relation to social adaptability and three domains of psychological difficulty among 34,097 secondary school students. After adjustment for sex, grade, and school clustering, both models contained direct and indirect associations whose bias-corrected bootstrap confidence intervals excluded zero. The magnitudes differed: attitudes toward parents had a total standardized association of −0.196 with psychological difficulties, whereas the total standardized association for co-residence was 0.049. Social adaptability was consistently associated with psychological difficulties (*β* = −0.491 and −0.505 across the two models). These estimates describe cross-sectional covariance patterns, not causal sequences.

### 6.2 Attitudes toward parents

Students who reported more positive attitudes toward their parents also reported greater social adaptability and fewer psychological difficulties. This pattern is consistent with literature linking adolescents’ perceptions of family cohesion or communication with adjustment (Cumsille and Epstein, 1994; Deniz et al., 2024). Nevertheless, the present item does not identify which aspect of the parent–child relationship is relevant. A high rating may reflect affection, low conflict, perceived support, current mood, or response style. The result therefore supports only a narrow conclusion about a global self-reported attitude.

The indirect estimate through social adaptability was larger than the residual direct estimate in the attitude model, but this decomposition does not demonstrate a psychological mechanism. It is equally possible that students with fewer psychological difficulties evaluate their family relationships more positively and feel more capable in social situations. Repeated measurements and more specific family constructs are needed to distinguish these alternatives.

### 6.3 Co-residence with parents

Students living with others rather than both parents reported slightly lower social adaptability and slightly greater psychological difficulties. The standardized total association was 0.049, indicating a small difference despite precise confidence intervals in the large sample. This result should not be translated into the claim that parental co-residence independently protects mental health. Co-residence does not identify parenting quality, continuing contact with nonresident parents, caregiver support, or the reason for separation.

Research on parental migration helps explain why the combined comparison is limited. Average associations between parental migration and child mental health have been reported (Fellmeth et al., 2018; Tang et al., 2018), but school engagement, economic resources, caregiver relationships, and the identity of the migrant parent may alter children’s experiences (Wen and Lin, 2012; Zhao et al., 2018). In the current dataset, paternal-grandparent, maternal-grandparent, and other arrangements could not be separated. The small binary association may conceal different patterns within these subgroups.

### 6.4 Social adaptability and practical implications

Social adaptability showed the largest standardized association with psychological difficulties in both models. This finding identifies a potentially relevant target for longitudinal research, but it does not show that increasing adaptability will reduce academic anxiety, self-blame, or impulsivity. The two constructs were measured concurrently and by the same reporter, so reciprocal relations and shared response variance are plausible.

Schools may reasonably assess students’ adjustment difficulties alongside family and mental health concerns, but program recommendations require evidence beyond this study. A targeted intervention has reported changes in social adaptability among adolescents receiving treatment for internet addiction (Zhao and Pan, 2022); that clinical context does not establish effectiveness in a general school population. Longitudinal studies should first test temporal ordering, followed by controlled trials of clearly specified school or family programs.

## 7 Strengths and limitations

The study’s strengths include a large regional sample, transparent separation of the two family-related indicators, continuous outcome analysis, school-level ICC estimation, and cluster-bootstrap confidence intervals for direct, indirect, and total associations. Reporting standardized and unstandardized estimates also helps distinguish statistical precision from substantive magnitude.

Several limitations remain. First, the cross-sectional design cannot establish temporal order or causal mediation (Maxwell and Cole, 2007). Second, all focal variables were reported by students in the same survey, creating possible common-method and response-style bias. Third, the two family-related predictors were single items. Attitudes toward parents cannot represent multidimensional relationship quality, and binary co-residence cannot represent family structure or functioning.

Fourth, the original four-category living-arrangement responses were not retained in the deidentified analytic file. The requested comparison of paternal-grandparent, maternal-grandparent, and other arrangements could not be conducted. Fifth, the outcome combined three selected MHT subscales rather than the complete instrument. Internal consistency was high, but the 35-item composite has not been independently validated as a short form, and its factor structure was not tested in the present analysis. The findings are therefore limited to difficulties related to academic anxiety, self-blame, and impulsivity.

Sixth, the social-adaptability scale was developed more than two decades ago. Its original categorical interpretations were not treated as current secondary-school norms, and confirmatory factor analysis was not conducted. Seventh, only sex and grade were available as covariates. Socioeconomic status, parental education, household income, urban or rural residence, family conflict, parental mental health, academic performance, only-child status, and parental migration may confound the observed associations (Conger et al., 2010).

Eighth, questionnaires were administered by class, but class identifiers were not retained. School-level clustering was addressed, yet class- and school-level variance could not be separated. The analysis also relied on 20 unequally sized school clusters, so cluster-level uncertainty estimates should be interpreted with care. Ninth, the archived records did not preserve the exact completion-time threshold or an exclusion-reason breakdown for invalid questionnaires. Finally, the sample came from one district of Chongqing; the large sample does not ensure generalizability to other Chinese regions or cultural settings.

## 8 Conclusion

More positive attitudes toward parents and co-residence with both parents were associated with greater social adaptability and fewer psychological difficulties in this regional cross-sectional sample. The association for co-residence was small, whereas attitudes toward parents and social adaptability showed larger associations. The indirect estimates were statistically compatible with pathways through social adaptability, but they do not establish causal mediation. Replication with longitudinal data, multidimensional family measures, updated measurement validation, richer covariates, and distinguishable living arrangements is needed before drawing mechanistic or intervention conclusions.

## Data Availability

The raw data supporting the conclusions of this article will be made available by the authors, without undue reservation.
